# ﻿Three new species of cave-adapted pseudoscorpions (Pseudoscorpiones, Chthoniidae) from eastern Yunnan, China

**DOI:** 10.3897/zookeys.1153.99537

**Published:** 2023-03-15

**Authors:** Yanmeng Hou, Zegang Feng, Feng Zhang

**Affiliations:** 1 The Key Laboratory of Zoological Systematics and Application, Institute of Life Science and Green Development, College of Life sciences, Hebei University, Baoding, Hebei 071002, China Hebei University Hebei China; 2 Key Laboratory of Zoological Systematics and Evolution, Institute of Zoology, Chinese Academy of Sciences, Beijing 100101, China Institute of Zoology, Chinese Academy of Sciences Beijing China; 3 College of Life Sciences, University of Chinese Academy of Sciences, Beijing, 100049, China University of Chinese Academy of Sciences Beijing China

**Keywords:** Cavernicolous, karst caves, *Lagynochthonius*, taxonomy, *Tyrannochthonius*

## Abstract

Three new cave-adapted chthoniid pseudoscorpions from four karst caves of Yunnan Province (China) are described, including detailed diagnosis and illustrations: *Tyrannochthoniuscalvatus***sp. nov.** from an unnamed cave and Dongtianfu Cave (Fuyuan County), *T.capito***sp. nov.** from Xianren Cave (Xichou County), and *Lagynochthoniusdaidaiensis***sp. nov.** from Daidai Cave (Qiubei County). All three species are endemic to Yunnan. *Tyrannochthoniuscalvatus***sp. nov.**, lacking the carapaceal antero-median setae and having intercalary teeth on the movable chelal finger only, is a peculiar chthoniid species.

## ﻿Introduction

The genus *Tyrannochthonius* Chamberlin, 1929 contains 149 species and two subspecies, with at least 58 species occurring in caves, and is distributed in all continents except Antarctica (Li 2022; WPC 2022). This genus can be diagnosed as follows: trichobothrium *sb* situated midway between *st* and *b*, or closer to *st*; trichobothria *ib* and *isb* situated close together in a median or sub-basal position on the dorsum of the chelal hand; chelal hand not distally constricted and the movable finger without a complex or strongly sclerotized apodeme at the base; fixed finger usually with one large, medial acuminate spine-like seta at its base, but can be reduced or absent in some cave-dwelling species; coxal spines generally long and present on coxae II only; epistome pointed, triangular or rounded, inconspicuous and usually with two closely-flanking setae at its base (Chamberlin 1962; Muchmore 1984, 1991; Muchmore and Chamberlin 1995; Edward and Harvey 2008). So far, 15 species and one subspecies of this genus have been described from China, of which 12 are exclusively known from karst caves (Mahnert 2009; Gao et al. 2018, 2020; Hou et al. 2022a; Li 2022; WPC 2022).

The genus *Lagynochthonius* Beier, 1951 was erected by Beier (1951) as a subgenus of *Tyrannochthonius* but was later elevated to generic status by Chamberlin (1962). The genus is diagnosed by trichobothrium *sb* situated midway between *st* and *b*, or closer to *st*; trichobothria *ib* and *isb* situated close together in a median or sub-basal position on the dorsum of the chelal hand; coxal spines generally long and present on coxae II only; chelal hand distally constricted (or flask-shaped) and movable finger with complex or strongly sclerotized apodeme at its base and the modified tooth (*td*) of the fixed chelal finger displaced onto the dorso-antiaxial face (Chamberlin 1962; Harvey 1989; Muchmore 1991; Judson 2007; Edward and Harvey 2008). At present, this genus contains 67 species (19 species living in caves) distributed in Asia, Australia, Africa, and America. Twenty species of this genus have been described from China, 13 of which are exclusively known from karst caves (Li et al. 2019; Hou et al. 2022a, b; WPC 2022).

Yunnan, located in southwest China, was once an ancient shallow sea during the Sinian (= Ediacaran) to Triassic periods and the area is characterized by massive karst landforms today (11.09 × 10^4^ km^2^) (Wang 2001). The influence of subtropical and tropical monsoon climates as well as the presence of rivers and precipitation regimes have probably fostered the development of karst caves. According to a survey, more than 1000 karst caves have been found in Yunnan Province (Ming 1997). To date, more than 750 cave-dwelling animals have been identified in China (nearly 15% of them are from Yunnan), including 54 cave-dwelling pseudoscorpion species (25 of them are from Yunnan) (Schawaller 1995; Mahnert 2003, 2009; Mahnert and Li 2016; Gao et al. 2017, 2018, 2020; Li et al. 2017, 2019; Feng et al. 2019, 2020; Latella 2019; Zhang et al. 2020; Li and Wang 2021; Hou et al. 2022a, b; Li 2022; WPC 2022; Xu et al. 2022).

Three new cavernicolous species of Chthoniidae have been recently found from the karst caves survey in Yunnan in 2021 and are here described.

## ﻿Materials and methods

The specimens examined for this study were cleared with a fine, soft-bristle brush and preserved in 75% alcohol and deposited in the Museum of Hebei University (**MHBU**) (Baoding, China) and the Museum of Southwest University (**MSWU**) (Chongqing, China). Photographs, drawings, and measurements were taken using a Leica M205A stereo-microscope equipped with a Leica DFC550 camera and the Inkscape software (v. 1.0.2.0). Detailed examination was carried out with an Olympus BX53 general optical microscope. All images were edited and formatted using Adobe Photoshop 2022.

Terminology and measurements follow Chamberlin (1931) with some minor modifications to the terminology of trichobothria (Harvey 1992; Judson 2007) and chelicera (Judson 2007). The chela and legs are measured in lateral view and others are taken in dorsal view. All measurements are given in mm unless noted otherwise. Proportions and measurements of chelicerae, carapace and pedipalps correspond to length/breadth, and those of legs, chela, and hand to length/depth.

The following abbreviations are used in the text: ***b*** basal trichobothrium; ***sb*** sub-basal trichobothrium; ***st*** sub-terminal trichobothrium; ***t*** terminal trichobothrium trichobothrium; ***ib*** interior basal trichobothrium; ***isb*** interior sub-basal trichobothrium; ***ist*** interior sub-terminal trichobothrium; ***it*** interior terminal trichobothrium; ***eb*** exterior basal trichobothrium; ***esb*** exterior sub-basal trichobothrium; ***est*** exterior sub-terminal trichobothrium; ***et*** exterior terminal trichobothrium; ***dx*** duplex trichobothria; ***sc*** microsetae (chemosensory setae); ***td*** modified tooth.

## ﻿Taxonomy

### ﻿Family Chthoniidae Daday, 1889

**Subfamily Chthoniinae Daday, 1889**

**Tribe Tyrannochthoniini Chamberlin, 1962**

#### ﻿Genus Tyrannochthonius

Taxon classificationAnimaliaPseudoscorpionesChthoniidae

Chamberlin, 1929

61A83093-AB70-509D-B9FF-B84CDF79297E

##### Type species.

*Chthoniusterribilis* With, 1906, by original designation.

#### ﻿ Tyrannochthonius calvatus sp. nov.

Taxon classificationAnimaliaPseudoscorpionesChthoniidae

EA6A9783-425E-5DD5-9F94-9D1620536FEF

https://zoobank.org/789C8A08-776E-47A8-BCC3-B73E4940FB3D

Figs 2 , 3 , 4 , 5 Chinese name: 秃头暴伪蝎

##### Type material.

**Figure 1.**
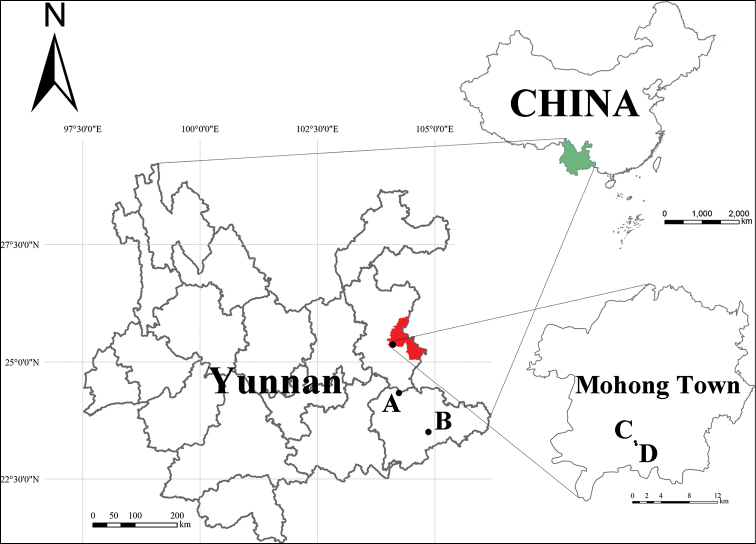
Study area, general cave locations, and type locality for each species, Yunnan Province, China. Each color represents an administrative region (green: Yunnan Province; red: Fuyuan County) **A** Daidai Cave (*Lagynochthoniusdaidaiensis* sp. nov.) **B** Xianren Cave (*Tyrannochthoniuscapito* sp. nov.) **C, D** unnamed cave and Dongtianfu Cave (*T.calvatus* sp. nov.).

**Figure 2.**
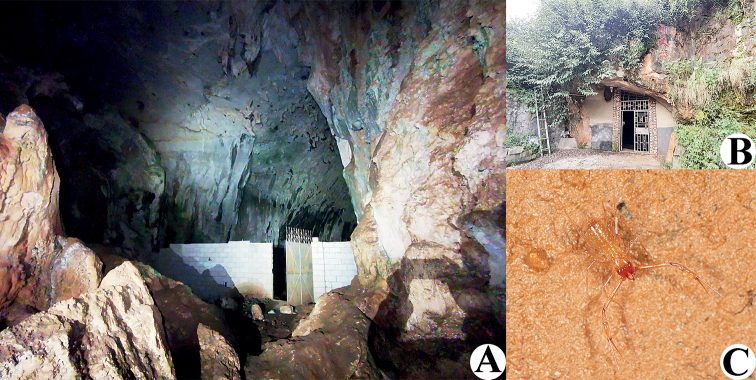
Unnamed cave (type locality) and Dongtianfu Cave, habitats of *Tyrannochthoniuscalvatus* sp. nov. **A** unnamed cave **B** Dongtianfu Cave **C** live female of *T.calvatus* sp. nov. in its natural environment from Dongtianfu Cave.

***Holotype***: China • ♂; Yunnan Province, Fuyuan County, Mohong Town, Puchong Village, unnamed cave; 25°22.301'N, 104°6.380'E; 2060 m a.s.l.; 07 Oct. 2021; Zegang Feng, Yanmeng Hou, Lu Zhang and Liu Fu leg.; under a stone in the dark zone; Ps.-MHBU-HBUARA#2021-429-01 (Figs 1C, 2A). ***Paratypes***: • 4 ♂; the same data as the holotype; Ps.-MHBU-HBUARA#2021-429-02-HBUARA#2021-429-05 • 1 ♂, 2 ♀; the same collection date and collectors as the holotype; Puchong Village, Dongtianfu Cave; 25°22.105'N, 104°6.447'E; 2035 m a.s.l.; under stones and clods in the dark zone; Ps.-MSWU-HBUARA#2021-428-01-HBUARA#2021-428-03 (Figs 1D, 2B).

##### Diagnosis

**(♂♀).** Moderately sized troglomorphic species with elongated appendages; carapace without eyes or eyespots; anterior margin of carapace thin, finely denticulated, with four setae (including preocular setae) only, without epistome and flanking basal setae; posterior margin of carapace with two setae; tergites I–III each with two setae. Pedipalps slender, femur 8.06–8.44 (♂), 7.94–8.38 (♀) × longer than broad; chela 8.05–8.33 (♂), 8.05–8.71 (♀) × longer than deep only movable chelal finger with intercalary teeth; movable chelal finger teeth markedly smaller than fixed chelal finger teeth and strongly retrorse and contiguous.

##### Etymology.

The specific name is derived from the Latin adjective *calvatus* (bald) and refers to the absence of two antero-median setae on the carapace.

##### Description.

**Figure 3.**
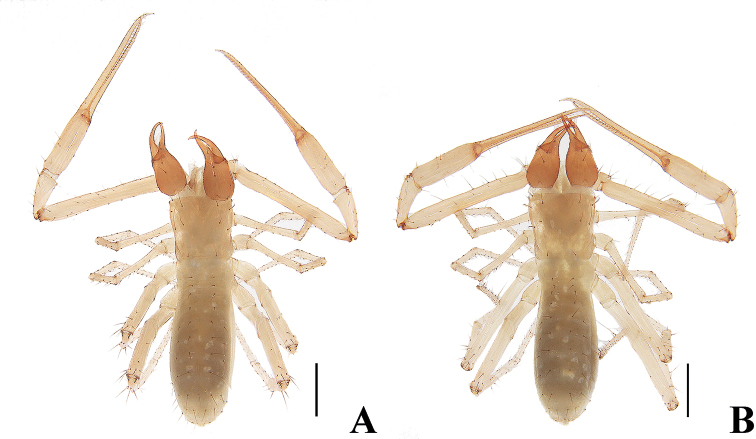
*Tyrannochthoniuscalvatus* sp. nov. **A** holotype male, habitus (dorsal view) **B** paratype female, habitus (dorsal view). Scale bars: 0.50 mm.

**Figure 4.**
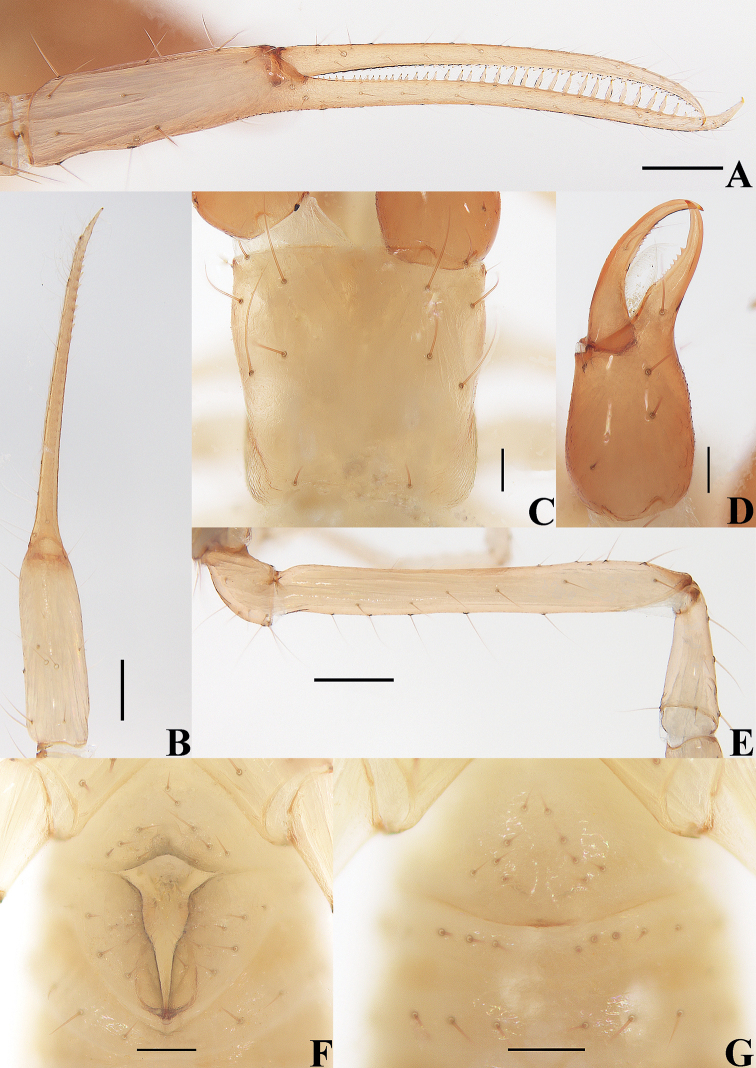
*Tyrannochthoniuscalvatus* sp. nov., holotype male (**A–F**), paratype female (**G**) **A** left chela (lateral view) **B** left chela (dorsal view) **C** carapace (dorsal view) **D** left chelicera (dorsal view) **E** left pedipalp (minus chela, dorsal view) **F** male genital area (ventral view) **G** female genital area (ventral view). Scale bars: 0.25 mm (**E**); 0.20 mm (**A, B**); 0.10 mm (**C, D, F, G**).

**Figure 5.**
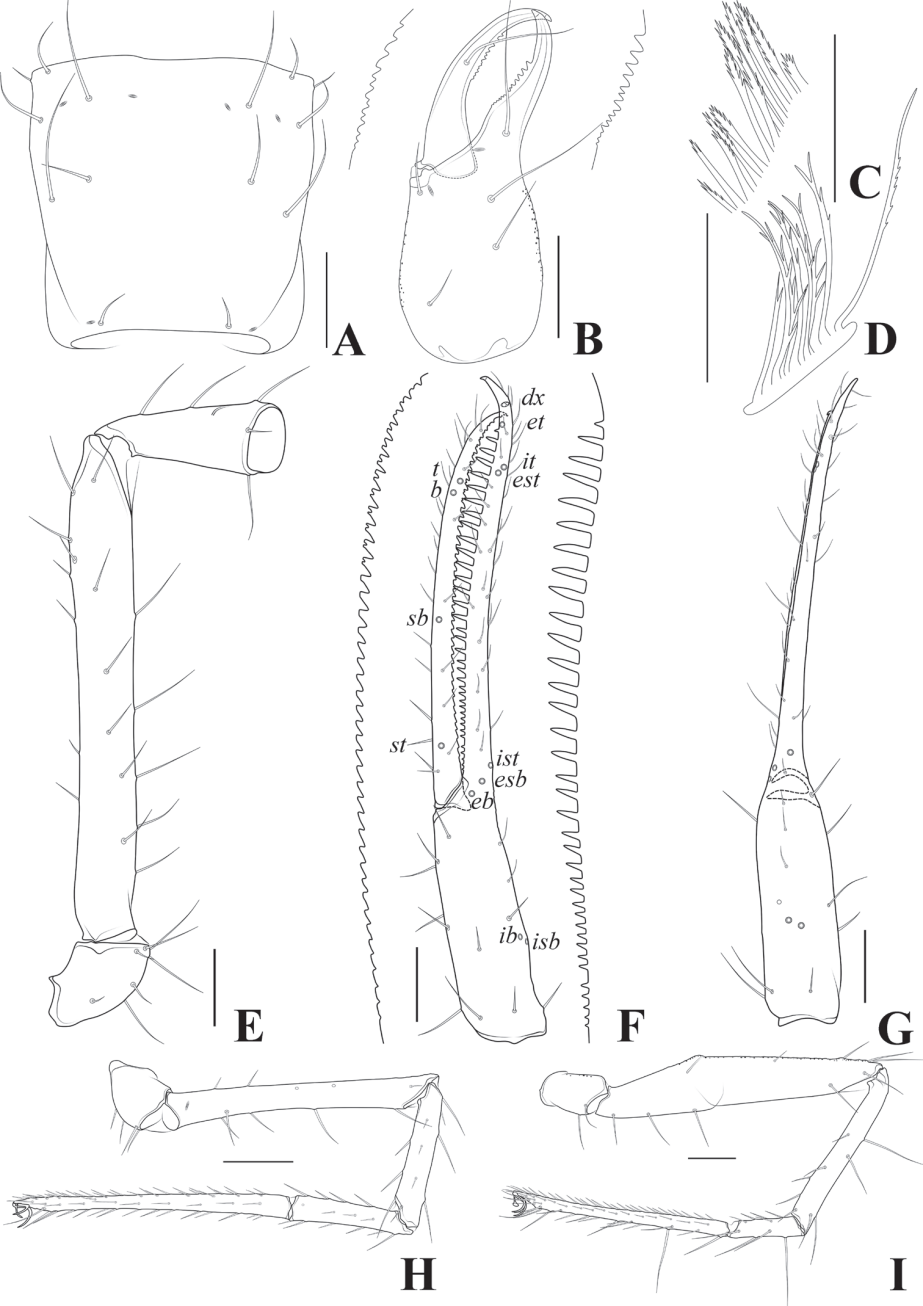
*Tyrannochthoniuscalvatus* sp. nov., holotype male **A** carapace (dorsal view) **B** left chelicera (dorsal view) with details of dentation **C** coxal spines on coxae II (ventral view) **D** rallum **E** left pedipalp (minus chela, dorsal view) **F** left chela (lateral view) with details of dentation and trichobothrial pattern **G** left chela (dorsal view) **H** leg I (lateral view) **I** leg IV (lateral view). Scale bars: 0.20 mm (**A, B, E–I**); 0.10 mm (**C, D**).

**Adult males** (Figs 3A, 4A–F, 5). ***Color***: generally pale yellow, chelicerae, pedipalps and tergites slightly darker, soft parts pale. ***Cephalothorax*** (Figs 4C, 5A): carapace 0.98× longer than broad, gently narrowed posteriorly; surface smooth, without furrows; no traces of eyes; anterior margin slightly serrate; epistome absent, without two setae flanking base; with 14–15 setae arranged s2s:4:4:0–1:2 (with 14–17 setae arranged s2s:4:4:0–3:2 in females), most setae heavy, long and gently curved, anterolateral setae much shorter than others; with three pairs of lyrifissures, the first two pairs situated middle and lateral to the setae of ocular row respectively, the third situated exterior to the sole pair of setae of posterior row. Chaetotaxy of coxae: P 3, I 3, II 3–4, III 5, IV 5; manducatory process with two acuminate distal setae, anterior seta more than 1/2 length of medial seta; apex of coxa I with small, rounded anteromedial process; coxae II with 12 or 13 terminally indented coxal spines on each side, set as an oblique and arc row, longer spines present in the middle of the row, becoming shorter distally and proximally and incised for ca. half their length (Fig. 5C); intercoxal tubercle absent; without sub-oral seta. ***Chelicera*** (Figs 4D, 5B, D): large, ca. as long as carapace, 2.43–2.48× longer than broad; five setae and two lyrifissures (exterior condylar lyrifissure and exterior lyrifissure) present on hand, all setae acuminate, ventrobasal seta shorter than others; movable finger with one medial seta. Cheliceral palm with moderate hispid granulation on both ventral and dorsal sides. Both fingers well provided with teeth, fixed finger with 14–16 teeth, distal one largest; movable finger with 13–15 retrorse contiguous small teeth; galea absent (Fig. 5B). Serrula exterior with 25–28 blades and serrula interior with 17 or 18 blades. Rallum with eight blades, the distal one longest and recumbent basally, with fine barbules and slightly set apart from the other blades, latter tightly grouped and with long pinnae, some of which are subdivided (Fig. 5D). ***Pedipalp*** (Figs 4A, B, E, 5E–G): long and slender, trochanter 1.74–1.88, femur 8.06–8.44, patella 2.37–2.61, chela 8.05–8.33, hand 3.00–3.10× longer than deep femur 2.87× longer than patella; movable chelal finger 1.64–1.66× longer than hand and 0.61–0.62× longer than chela. Setae generally long and acuminate; one distal lyrifissure present on patella (Figs 4E, 5E). Chelal palm not constricted towards fingers, apodeme complex of movable chelal finger only slightly sclerotized, with weak granulation dorsally at base of fixed chelal finger. Fixed chelal finger and hand with eight trichobothria, movable chelal finger with four trichobothria, *ib* and *isb* situated close together, submedially on dorsum of chelal hand; *eb*, *esb*, and *ist* forming a nearly straight oblique row at base of fixed chelal finger; *it* slightly distal to *est*, situated subdistally; *et* slightly near to tip of fixed chelal finger, very close to chelal teeth; *dx* situated distal to *et*; *sb* situated midway between *b* and *st*; *b* and *t* situated subdistally, *t* situated distal to *b* and proximal to *est* (Fig. 5F). A tiny antiaxial lyrifissure present at base of fixed chelal finger (situated distal to *ist*). Both chelal fingers with a row of teeth, heterodentate, spaced regularly along the margin, larger and well-spaced teeth present in the middle of the row, becoming smaller and closer distally and proximally: fixed chelal finger with 32–36 macrodenticles, long and pointed, without intercalary teeth; movable chelal finger with 37–41 macrodenticles (smaller than teeth on fixed chelal finger), retrorse and contiguous, plus 10–12 intercalary microdenticles (roughly extending to *b*), 47–53 in total (Fig. 5F). Chelal fingers straight in dorsal view; microsetae (*sc*) present on dorsum of chelal hand (Figs 4B, 5G). ***Opisthosoma***: generally typical, pleural membrane finely granulated. Tergites and sternites undivided; setae uniseriate and acuminate. Tergal chaetotaxy I–XII: 2:2:2:2:4:4:4:4:4–5:4:T2–3T:0; tergite IX with an unpaired median seta. Sternal chaetotaxy III–XII: 12–14:11–12:8:9:9:9–10:9:7–9:0: 2. Anterior genital operculum with ten setae, genital opening slit-like, with 10–12 marginal setae on each side, 31–33 in total (Fig. 4F). ***Legs*** (Fig. 5H, 5I): generally typical, long and slender. Fine granulation present on anterodorsal faces of femur IV and patella IV. Femur of leg I 1.88× longer than patella and with one lyrifissure at the base of femur; tarsus 2.45× longer than tibia. Femoropatella of leg IV 4.83–5.04× longer than deep; tibia 7.60× longer than deep; with basal tactile setae on both tarsal segments: basitarsus 4.00–4.25× longer than deep (TS = 0.24–0.28), telotarsus 15.33–15.50× longer than deep and 2.71–2.91× longer than basitarsus (TS = 0.27–0.28). Arolium slightly shorter than the claws, not divided; claws simple.

**Adult females** (Figs 2C, 3B, 4G). Mostly same as males, but a little larger; chaetotaxy of coxae: P 3, I 3, II 4, III 5, IV 5; tergal chaetotaxy I–XII: 2:2:2:2–3:4:4:3–4:4:4–5:4:T2T:0; sternal chaetotaxy IV–XII: 10–12:9–10:7–10:9:9:9:6–8:0:2; anterior genital operculum with 10–13 setae, posterior margin with 10–14 marginal setae, 22–24 in total; leg IV with a long tactile seta on both tarsal segments: basitarsus 3.56–5.00× longer than deep (TS = 0.23–0.25), telotarsus 14.00–16.17× longer than deep and 2.77–2.88× longer than basitarsus (TS = 0.27–0.29).

***Dimensions*** (length/breadth or, in the case of the legs, also for chela and hand, length/depth in mm). Males (females in parentheses): body length 2.16–2.21 (2.13–2.26). Pedipalps: trochanter 0.32–0.33/0.17–0.19 (0.32–0.34/0.18–0.20), femur 1.29–1.35/0.16 (1.34–1.37/0.16–0.17), patella 0.45–0.47/0.18–0.19 (0.49–0.52/0.18–0.20), chela 1.75–1.77/0.21–0.22 (1.77–1.84/0.21–0.22), hand 0.65–0.66/0.21–0.22 (0.65–0.68/0.21–0.22), movable chelal finger length 1.08 (1.11–1.16). Chelicera 0.72–0.73/0.29–0.30 (0.73–0.76/0.29–0.32), movable finger length 0.40 (0.40–0.41). Carapace 0.59/0.59–0.60 (0.59–0.65/0.62–0.66). Leg I: trochanter 0.21/0.13–0.14 (0.20–0.22/0.12–0.16), femur 0.75–0.77/0.09 (0.77–0.82/0.08–0.10), patella 0.40–0.41/0.08 (0.41–0.44/0.07–0.09), tibia 0.33/0.07 (0.34–0.37/0.06–0.07), tarsus 0.81/0.06 (0.79–0.82/0.06–0.07). Leg IV: trochanter 0.29–0.32/0.18 (0.28–0.30/0.15–0.18), femoropatella 1.11–1.16/0.23 (1.11–1.15/0.20–0.24), tibia 0.76/0.10 (0.77–0.81/0.10), basitarsus 0.32–0.34/0.08 (0.32–0.35/0.07–0.09), telotarsus 0.92–0.93/0.06 (0.92–0.98/0.06–0.07).

##### Remarks.

*Tyrannochthoniuscalvatus* sp. nov. can be easily distinguished from other Chinese cave-dwelling *Tyrannochthonius* species by lacking two carapaceal antero-median setae and the presence of intercalary teeth on the movable chelal finger only.

##### Distribution.

Known only from the unnamed cave (type locality) and Dongtianfu Cave.

#### ﻿ Tyrannochthonius capito sp. nov.

Taxon classificationAnimaliaPseudoscorpionesChthoniidae

2D2DC9F4-9853-59EE-8ECA-B04B3B046AFD

https://zoobank.org/9D5EEEEF-13A3-4C9C-895D-897EC8C8DF0E

**Figure 6.**
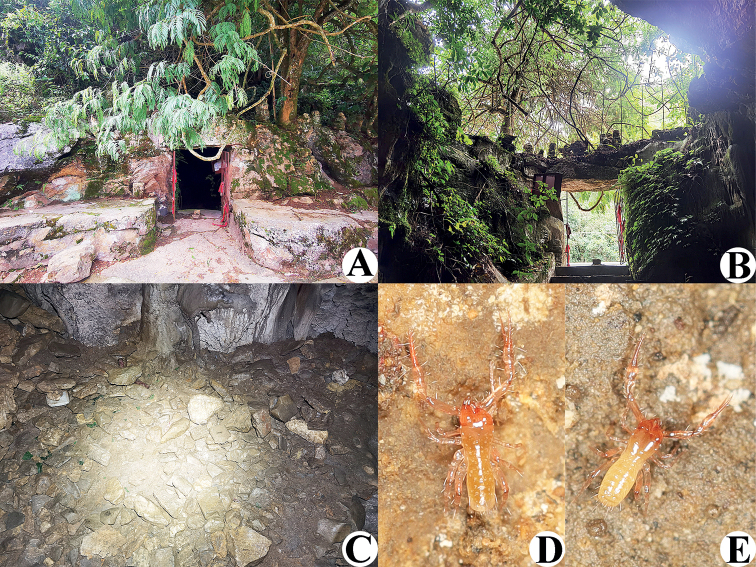
Xianren Cave (type locality), habitat of *Tyrannochthoniuscapito* sp. nov. **A** entrance **B** inside the cave entrance **C** area where *T.capito* sp. nov. specimens were collected **D** live male of *T.capito* sp. nov. in its natural environment **E** live female of *T.capito* sp. nov. in its natural environment.

**Figure 7.**
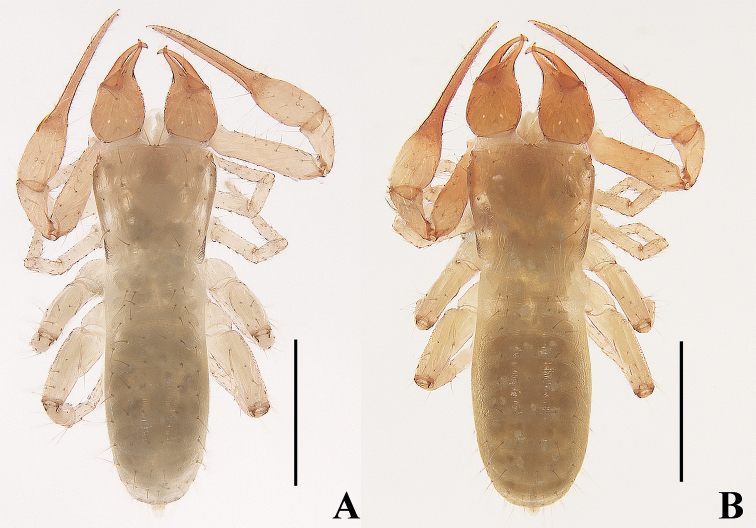
*Tyrannochthoniuscapito* sp. nov. **A** holotype male, habitus (dorsal view) **B** paratype female, habitus (dorsal view). Scale bars: 0.50 mm.

**Figure 8.**
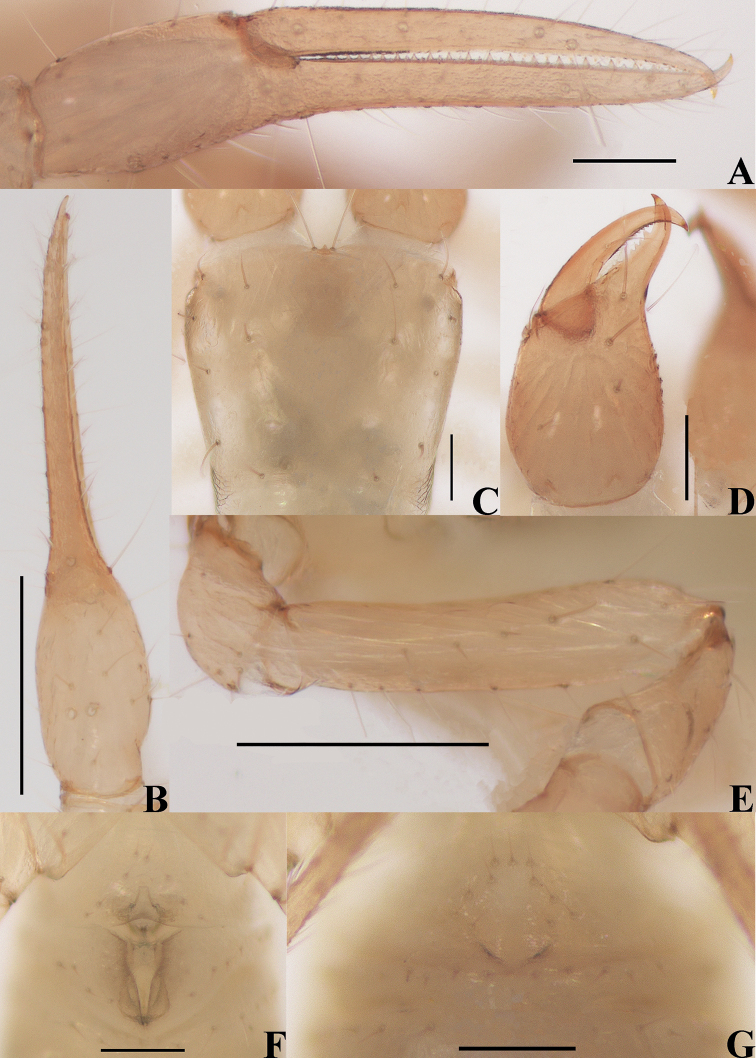
*Tyrannochthoniuscapito* sp. nov., holotype male (**A–F**), paratype female (**G**) **A** left chela (lateral view) **B** left chela (dorsal view) **C** carapace (dorsal view) **D** left chelicera (dorsal view) **E** left pedipalp (minus chela, dorsal view) **F** male genital area (ventral view) **G** female genital area (ventral view). Scale bars: 0.25 mm (**B, E**); 0.10 mm (**A, C, D, F, G**).

**Figure 9.**
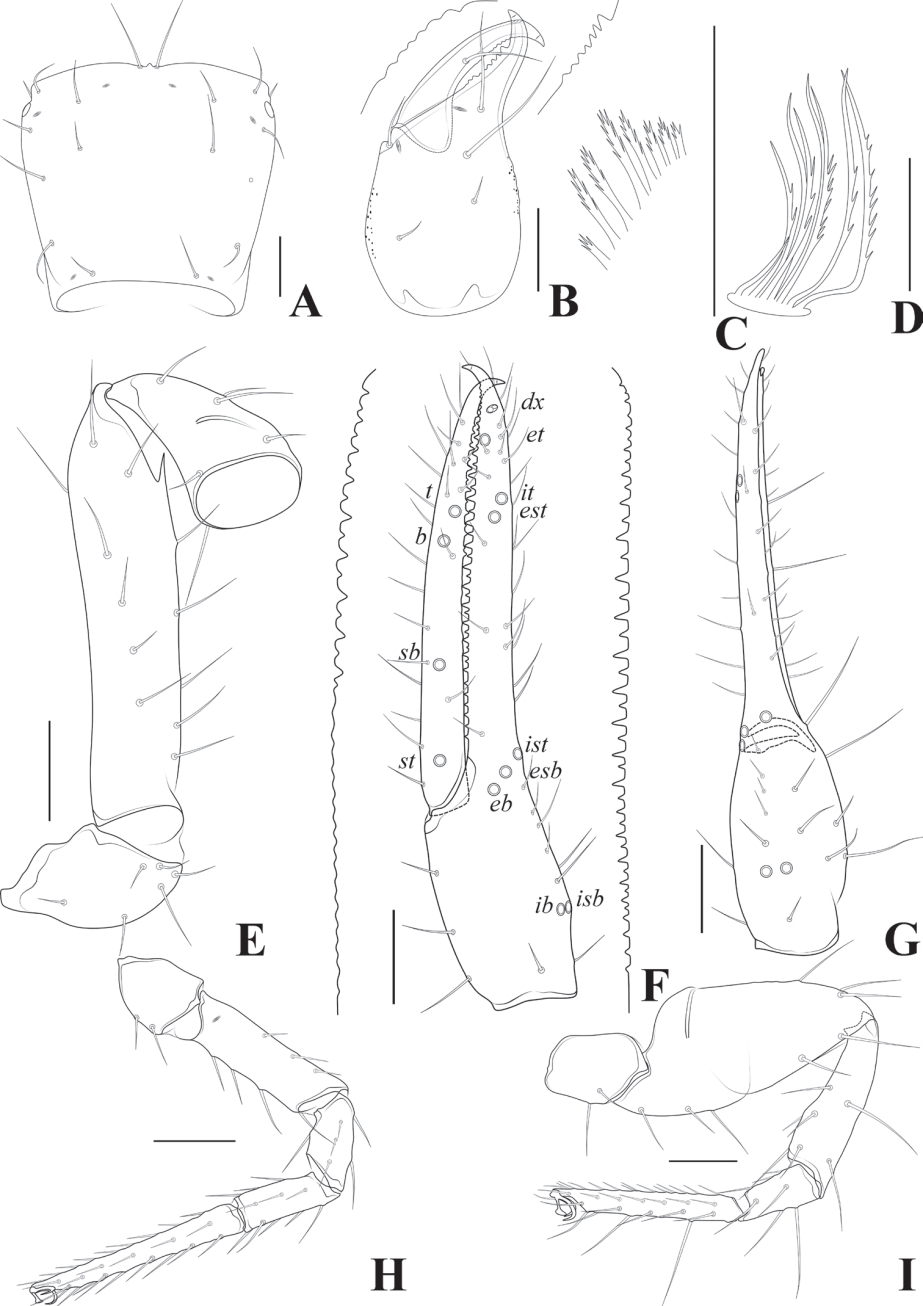
*Tyrannochthoniuscapito* sp. nov., holotype male **A** carapace (dorsal view) **B** left chelicera (dorsal view) with details of dentation **C** coxal spines on coxae II (ventral view) **D** rallum **E** left pedipalp (minus chela, dorsal view) **F** left chela (lateral view) with details of dentation and trichobothrial pattern **G** left chela (dorsal view) **H** leg I (lateral view) **I** leg IV (lateral view). Scale bars: 0.10 mm.

Figs 6 , 7 , 8 , 9 Chinese name: 大头暴伪蝎

##### Type material.

***Holotype***: China • ♂; Yunnan Province, Xichou County, Jijie Township, Xianrendong Village, Xianren Cave; 23°30.124'N, 104°52.082'E; 1345 m a.s.l.; 16 Oct. 2021; Zegang Feng, Yanmeng Hou, Lu Zhang and Liu Fu leg.; under a stone in the dark zone; Ps.-MHBU-HBUARA#2021-443-01 (Figs 1B, 6). ***Paratypes***: • 1 ♂, 2 ♀; the same data as the holotype; Ps.-MSWU-HBUARA#2021-443-02-HBUARA#2021-443-04.

##### Diagnosis

**(♂♀).** Small-sized cavernicolous species with slightly elongated appendages; carapace with two anterior corneate eyes only; anterior margin of carapace thin, finely denticulated, epistome small, pointed, triangular; posterior margin of carapace with two setae; tergites I–III each with four setae. Pedipalps slender, femur 4.30–4.67 (♂), 4.27–4.60 (♀) × longer than broad; chela 4.77–5.23 (♂), 4.53–4.73 (♀) × longer than deep; both chelal fingers with intercalary teeth.

##### Etymology.

The specific name is derived from the Latin noun *capito* (big head) and refers to the presence of a large cephalothorax.

##### Description.

**Adult males** (Figs 6D, 7A, 8A–F, 9). ***Color***: generally pale yellow, chelicerae, pedipalps and tergites slightly darker, soft parts pale. ***Cephalothorax*** (Figs 8C, 9A): carapace 0.88–0.95× longer than broad, gently narrowed posteriorly; surface smooth, without furrows; with two anterior eyes only; anterior margin slightly serrate; epistome small, pointed, triangular; with 18 setae arranged s4s:4:4:2:2, most setae heavy, long and gently curved, anterolateral setae much shorter than others; with three pairs of lyrifissures, the first two pairs situated middle and lateral to the setae of ocular row respectively, the third situated exterior to the sole pair of setae of posterior row. Chaetotaxy of coxae: P 3, I 3–5, II 4, III 5, IV 5; manducatory process with two acuminate distal setae, anterior seta more than 1/2 length of medial seta; apex of coxa I with small, rounded anteromedial process; coxa II with seven or eight terminally indented coxal spines on each side, set as an oblique and arc row, longer spines present in the middle of the row, becoming shorter distally and proximally and incised for ca. half their length (Fig. 9C); intercoxal tubercle absent; without sub-oral seta. ***Chelicera*** (Figs 8D, 9B, D): large, ca. as long as carapace, 2.00× longer than broad; five setae and two lyrifissures (exterior condylar lyrifissure and exterior lyrifissure) present on hand, all setae acuminate, ventrobasal seta shorter than others; movable finger with one medial seta. Cheliceral palm with moderate hispid granulation on both ventral and dorsal sides. Both fingers well provided with teeth, fixed finger with seven or nine acute teeth, distal one largest; movable finger with seven or eight rounded and contiguous small teeth; galea represented by a very slight bump on movable finger (Fig. 9B). Serrula exterior with 19 or 20 blades and serrula interior with 16 blades. Rallum with eight blades, the distal one longest and recumbent basally, with fine barbules and slightly set apart from the other blades, latter tightly grouped and with long pinnae, some of which are subdivided (Fig. 9D). ***Pedipalp*** (Figs 8A, B, E, 9E–G): long and slender, trochanter 1.64–1.70, femur 4.30–4.67, patella 1.67–2.00, chela 4.77–5.23, hand 1.62–1.77× longer than broad; femur 2.10–2.15× longer than patella; movable chelal finger 2.00–2.04× longer than hand and 0.68–0.69× longer than chela. Setae generally long and acuminate; one distal lyrifissure present on patella (Figs 8E, 9E). Chelal palm not constricted towards fingers, apodeme complex of movable chelal finger only slightly sclerotized, with weak granulation dorsally at base of fixed chelal finger. Fixed chelal finger and hand with eight trichobothria, movable chelal finger with four trichobothria, *ib* and *isb* situated close together, submedially on dorsum of chelal hand; *eb*, *esb*, and *ist* forming a straight oblique row at base of fixed chelal finger; *it* slightly distal to *est*, situated subdistally; *et* slightly near to tip of fixed chelal finger, very close to chelal teeth; *dx* situated distal to *et*; *sb* situated closer to *st* than to *b*; *b* and *t* situated subdistally, *t* situated distal to *b* and at same level as *est* (Fig. 9F). A tiny antiaxial lyrifissure present at base of fixed chelal finger (situated distal to *ist*). Both chelal fingers with a row of teeth, heterodentate, spaced regularly along the margin, larger and well-spaced teeth present in the middle of the row, becoming smaller and closer distally and proximally: fixed chelal finger with 22 or 23 macrodenticles, pointed and slightly retrorse, plus 19 or 20 intercalary microdenticles, 42 in total; movable chelal finger with nine or ten macrodenticles (slightly smaller than teeth on fixed chelal finger), pointed and slightly retrorse, plus nine intercalary microdenticles and 17 or 18 vestigial, rounded and contiguous basal teeth, 36 in total (Fig. 9F). Chelal fingers straight in dorsal view; microsetae (*sc*) present on dorsum of chelal hand (Figs 8B, 9G). ***Opisthosoma***: generally typical, pleural membrane finely granulated. Tergites and sternites undivided; setae uniseriate and acuminate. Tergal chaetotaxy I–XII: 4:4:4:6:6:6:6:6:6:7:T2T:0. Sternal chaetotaxy III–XII: 13:12–14:10:9:9–10:10–12:11–12:10:0:2. Anterior genital operculum with 8 setae, genital opening slit-like, with 11 or 12 marginal setae on each side, 31 in total (Fig. 8F). ***Legs*** (Fig. 9H, I): generally typical, long and slender. Fine granulation present on anterodorsal faces of femur IV and patella IV. Femur of leg I 1.83–1.92× longer than patella and with one lyrifissure at the base of femur; tarsus 2.00–2.25× longer than tibia. Femoropatella of leg IV 1.89–2.20× longer than deep; tibia 3.25–3.71× longer than deep; with basal tactile setae on both tarsal segments: basitarsus 2.00–2.20× longer than deep (TS = 0.33–0.36), telotarsus 6.25–7.00× longer than deep and 2.27–2.33× longer than basitarsus (TS = 0.28–0.29). Arolium slightly shorter than the claws, not divided; claws simple.

**Adult females** (Figs 6E, 7B, 8G). Mostly same as males, but a little larger; chaetotaxy of coxae: P 3, I 3, II 4, III 5, IV 5; tergal chaetotaxy I–XII: 4:4:4:6:6–7:6:6–7:6:6–7:7:T2T:0; sternal chaetotaxy IV–XII: 12–13:9–11:9:9–11:12–13:13:11–13:0:2; anterior genital operculum with 10 or 11 setae, posterior margin with 14 marginal setae, 24 or 25 in total; leg IV with a long tactile seta on both tarsal segments: basitarsus 2.00–2.17× longer than deep (TS = 0.31–0.33), telotarsus 7.00–9.00× longer than deep and 2.08–2.33× longer than basitarsus (TS = 0.25–0.26).

***Dimensions*** (length/breadth or, in the case of the legs, also for chela and hand, length/depth in mm). Males (females in parentheses): body length 1.08–1.25 (1.31–1.37). Pedipalps: trochanter 0.17–0.18/0.10–0.11 (0.19–0.20/0.10), femur 0.42–0.43/0.09–0.10 (0.46–0.47/0.10–0.11), patella 0.20/0.10–0.12 (0.23–0.24/0.11–0.12), chela 0.62–0.68/0.13 (0.68–0.71/0.15), hand 0.21–0.23/0.13 (0.24/0.15), movable chelal finger length 0.42–0.47 (0.46–0.49). Chelicera 0.34–0.38/0.17–0.19 (0.40–0.41/0.20), movable finger length 0.19–0.21 (0.22–0.23). Carapace 0.37/0.39–0.42 (0.38–0.39/0.45–0.46). Leg I: trochanter 0.10–0.11/0.08–0.09 (0.11–0.12/0.08–0.09), femur 0.22–0.23/0.06 (0.25–0.26/0.07), patella 0.12/0.05 (0.13–0.14/0.06), tibia 0.12–0.15/0.04 (0.13–0.15/0.04), tarsus 0.27–0.30/0.03–0.04 (0.27–0.28/0.04). Leg IV: trochanter 0.14/0.10–0.11 (0.14–0.16/0.10–0.11), femoropatella 0.33–0.34/0.15–0.18 (0.36–0.38/0.14–0.16), tibia 0.26/0.07–0.08 (0.27/0.07–0.08), basitarsus 0.11–0.12/0.05–0.06 (0.12–0.13/0.06), telotarsus 0.25–0.28/0.04 (0.27–0.28/0.03–0.04).

##### Remarks.

*Tyrannochthoniuscapito* sp. nov. is similar to an epigean species *T.robustus* Beier, 1951 (from Vietnam and China) in having intercalary teeth on both chelal fingers and similar body size (e.g., body length 1.08–1.25 mm vs. 1.20 mm (♂), 1.31–1.37 mm vs. 1.20 mm (♀)), but differs by the number of eyes (2 vs. 4), the presence of a hypopigmented body cuticle and the ratio of movable chelal finger and chelal hand (2.00–2.04× vs. 1.52× (♂), 1.92–2.04× vs. 1.80× (♀)) (Beier 1951; Song 1996).

*Tyrannochthoniuscapito* sp. nov. can be easily distinguished from other Chinese cave-dwelling *Tyrannochthonius* species by the presence of a pair of anterior corneate eyes.

##### Distribution.

Known only from the type locality.

#### ﻿Genus Lagynochthonius

Taxon classificationAnimaliaPseudoscorpionesChthoniidae

Beier, 1951

D064F66B-CFA2-54D3-AF21-E78768CEAA9B

##### Type species.

*Chthoniusjohni* Redikorzev, 1922, by original designation.

#### ﻿ Lagynochthonius daidaiensis sp. nov.

Taxon classificationAnimaliaPseudoscorpionesChthoniidae

FE02CCC0-BBCB-5ECB-98A8-A3ACE836B8B6

https://zoobank.org/D23220D7-2AF6-47AE-B848-941BED5096E1

**Figure 10.**
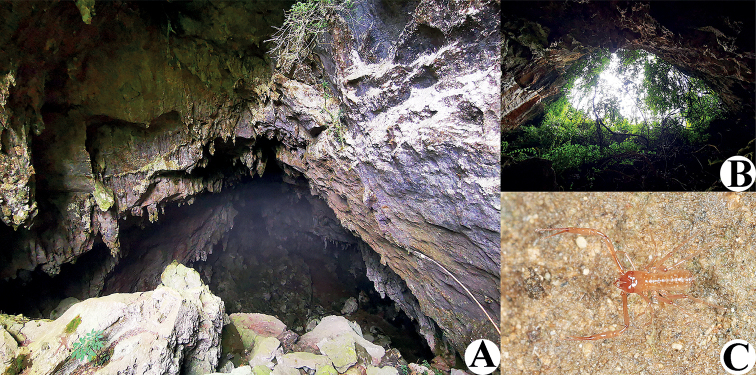
Daidai Cave (type locality), habitat of *Lagynochthoniusdaidaiensis* sp. nov. **A** entrance **B** inside the cave entrance **C** live female of *L.daidaiensis* sp. nov. in its natural environment.

**Figure 11.**
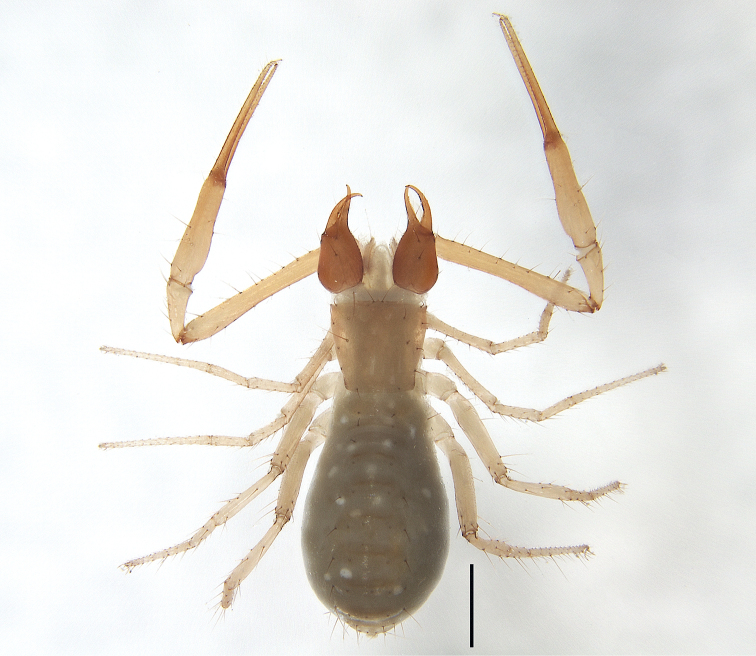
*Lagynochthoniusdaidaiensis* sp. nov., holotype female, habitus (dorsal view). Scale bar: 0.50 mm.

**Figure 12.**
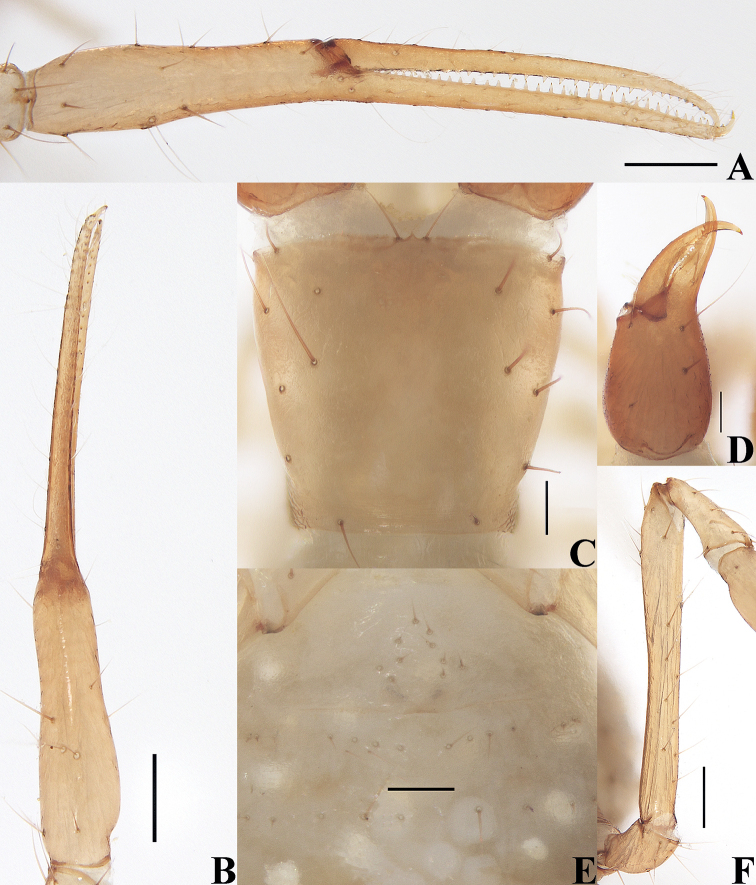
*Lagynochthoniusdaidaiensis* sp. nov., holotype female **A** left chela (lateral view) **B** left chela (dorsal view) **C** carapace (dorsal view) **D** left chelicera (dorsal view) **E** female genital area (ventral view) **F** left pedipalp (minus chela, dorsal view). Scale bars: 0.20 mm (**A, B, F**); 0.10 mm (**C–E**).

**Figure 13.**
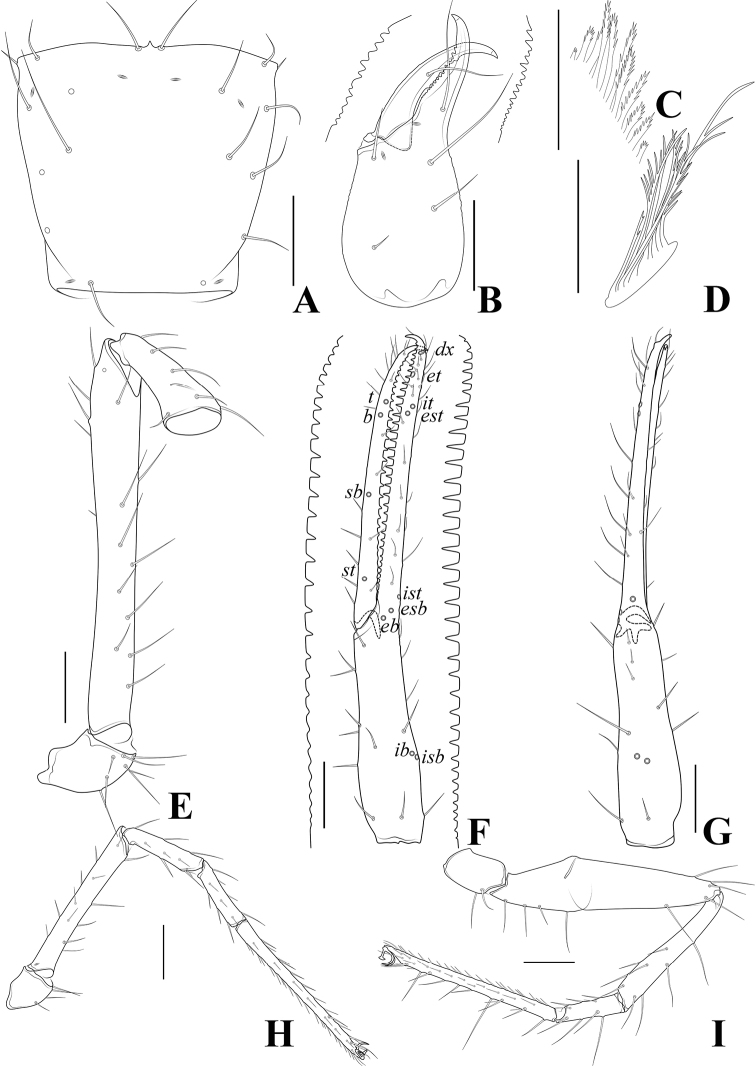
*Lagynochthoniusdaidaiensis* sp. nov., holotype female **A** carapace (dorsal view) **B** left chelicera (dorsal view) with details of dentation **C** coxal spines on coxae II (ventral view) **D** rallum **E** left pedipalp (minus chela, dorsal view) **F** left chela (lateral view) with details of dentation and trichobothrial pattern **G** left chela (dorsal view) **H** leg I (lateral view) **I** leg IV (lateral view). Scale bars: 0.20 mm (**A, B, E–I**); 0.10 mm (**C, D**).

Figs 10 , 11 , 12 , 13 Chinese name: 呆呆拉伪蝎

##### Type material.

***Holotype***: China • ♀; Yunnan Province, Qiubei County, Shuanglongying Town, Pingtan Village, Daidai Cave; 24°19.772'N, 104°14.355'E; 1228 m a.s.l.; 20 Jul. 2021; Zegang Feng, Hongru Xu, Liu Fu and Nana Zhan leg.; under a stone in the dark zone; Ps.-MHBU-HBUARA#2021-176-01 (Figs 1A, 10). ***Paratypes***: • 2 ♀; the same data as the holotype; Ps.-MSWU-HBUARA#2021-176-02-HBUARA#2021-176-03.

##### Diagnosis

**(♀).** Moderately sized troglomorphic species with elongated appendages; carapace without eyes or eyespots; anterior margin of carapace thin, finely denticulated, epistome pointed and small, triangular; posterior margin of carapace with two setae; tergites I–IV each with two setae. Pedipalps slender, femur 7.79–8.07× longer than broad; chela 7.67–8.39× longer than deep; both chelal fingers without intercalary teeth but fixed chelal finger with a modified accessory tooth (*td*) on dorso-antiaxial face.

##### Etymology.

Named after the type locality, Daidai Cave.

##### Description.

**Adult females** (male unknown) (Figs 10C, 11–13). ***Color***: generally pale yellow, chelicerae, pedipalps and tergites slightly darker, soft parts pale. ***Cephalothorax*** (Figs 12C, 13A, C): carapace 0.97× longer than broad, gently narrowed posteriorly; surface smooth, without furrows; no traces of eyes; anterior margin slightly serrate; epistome pointed and small, triangular; with 18 setae arranged s4s:4:4:2:2, most setae heavy, long and gently curved, anterolateral setae much shorter than others; with three pairs of lyrifissures, the first two pairs situated middle and lateral to the setae of ocular row respectively, the third situated exterior to the sole pair of setae of posterior row. Chaetotaxy of coxae: P 3, I 3, II 3–4, III 5, IV 5; manducatory process with two acuminate distal setae, anterior seta less than 1/2 length of medial seta; apex of coxa I with small, rounded anteromedial process; coxae II with 11 or 12 terminally indented coxal spines on each side, set as an oblique and arc row, longer spines present in the middle of the row, becoming shorter distally and proximally and incised for ca. half their length (Fig. 13C); intercoxal tubercle absent; without sub-oral seta. ***Chelicera*** (Figs 12D, 13B, D): large, ca. as long as carapace, 2.41× longer than broad; five setae and two lyrifissures (exterior condylar lyrifissure and exterior lyrifissure) present on hand, all setae acuminate, ventrobasal seta shorter than others; movable finger with one medial seta. Cheliceral palm with moderate hispid granulation on both ventral and dorsal sides. Both fingers well provided with teeth, fixed finger with 15–17 teeth, distal one largest; movable finger with 12 retrorse contiguous small teeth; galea absent (Fig. 13B). Serrula exterior with 21 blades and serrula interior with 13 or 14 blades. Rallum with seven blades, the distal one longest and recumbent basally, with fine barbules and slightly set apart from the other blades, latter tightly grouped and with long pinnae, some of which are subdivided (Fig. 13D). ***Pedipalp*** (Figs 12A, B, F, 13E–G): long and slender, trochanter 1.25–1.41, femur 7.79–8.07, patella 2.35–2.53, chela 7.67–8.39, hand 3.29–3.61× longer than broad; femur 2.83–2.87× longer than patella; movable chelal finger 1.32–1.35× longer than hand and 0.57–0.58× longer than chela. Setae generally long and acuminate; one distal lyrifissure present on patella (Figs 12F, 13E). Chelal palm gradually constricted towards fingers, apodeme complex of movable chelal finger strongly sclerotized, with weak granulation dorsally at base of fixed chelal finger. Fixed chelal finger and hand with eight trichobothria, movable chelal finger with four trichobothria, *ib* and *isb* situated close together, submedially on dorsum of chelal hand; *eb*, *esb*, and *ist* forming an oblique row at base of fixed chelal finger; *it* slightly distal to *est*, situated subdistally; *et* slightly near to tip of fixed chelal finger, very close to chelal teeth; *dx* situated distal to *et*; *sb* situated midway between *b* and *st*; *b* and *t* situated subdistally, *t* situated distal to *b*; *est* and *it* situated between *b* and *t* (Fig. 13F). A tiny antiaxial lyrifissure present at base of fixed chelal finger (situated distal to *ist*). Both chelal fingers with a row of teeth, homodentate, spaced regularly along the margin, larger teeth present in the middle of the row, becoming smaller and closer distally and proximally: fixed chelal finger with 35 or 36 macrodenticles, slightly retrorse and pointed, plus a modified accessory tooth on dorso-antiaxial face (*td*, close to *dx*), 36 or 37 in total; movable chelal finger with 20–22 macrodenticles (slightly smaller than teeth on fixed chelal finger), slightly retrorse and pointed, plus nine or ten vestigial, rounded and contiguous basal teeth, 30 or 31 in total (Fig. 13F). Chelal fingers slightly curved in dorsal view; microsetae (*sc*) present on dorsum of chelal hand (Figs 12B, 13G). ***Opisthosoma***: generally typical, pleural membrane finely granulated. Tergites and sternites undivided; setae uniseriate and acuminate. Tergal chaetotaxy I–XII: 2:2:2:2:4:4–5:4–5:5:5:4:T2T:0, tergites VIII and IX each with an unpaired median seta. Sternal chaetotaxy IV–XII: 11–12:7–8:7:7–8:7–8:7:7–8:0:2. Anterior genital operculum with 10 setae, posterior margin with 13–14 marginal setae, 23–24 in total (Fig. 12E). ***Legs*** (Fig. 13H, I): generally typical, long and slender. Fine granulation present on anterodorsal faces of femur IV and patella IV. Femur of leg I 1.88–1.97× longer than patella and with one lyrifissure at the base of femur; tarsus 2.72–2.73× longer than tibia. Femoropatella of leg IV 4.00× longer than deep; tibia 6.20–6.33× longer than deep; with basal tactile setae on both tarsal segments: basitarsus 3.50–3.71× longer than deep (TS = 0.29–0.35), telotarsus 14.60–15.20× longer than deep and 2.71–2.81× longer than basitarsus (TS = 0.36). Arolium slightly shorter than the claws, not divided; claws simple. ***Dimensions of adult females*** (length/breadth or, in the case of the legs, also for chela and hand, length/depth in mm): body length 1.85–2.04. Pedipalps: trochanter 0.20–0.24/0.16–0.17, femur 1.09–1.13/0.14, patella 0.38–0.40/0.15–0.17, chela 1.51–1.61/0.18–0.21, hand 0.65–0.69/0.18–0.21, movable chelal finger length 0.86–0.93. Chelicera 0.65–0.70/0.27–0.29, movable finger length 0.34–0.36. Carapace 0.56–0.58/0.58–0.60. Leg I: trochanter 0.16–0.18/0.15, femur 0.60–0.65/0.08, patella 0.32–0.33/0.07, tibia 0.25–0.26/0.05–0.06, tarsus 0.68–0.71/0.05. Leg IV: trochanter 0.25–0.26/0.15, femoropatella 0.84–0.92/0.21–0.23, tibia 0.57–0.62/0.09–0.10, basitarsus 0.26–0.28/0.07–0.08, telotarsus 0.73–0.76/0.05.

##### Remarks.

*Lagynochthoniusdaidaiensis* sp. nov. is similar to *L.laoxueyanensis* Hou, Gao & Zhang, 2022 (from Yunnan, China), but differs by the number of setae on tergites III–IV (2 vs. 4), a shorter chela (chela 7.67–8.39 vs. 6.88–7.22 (♀) × longer than deep, length 1.51–1.61 vs. 1.65–1.66 mm) and the number of coxal spines blades (11 or 12 vs. 9) (Hou et al. 2022a).

*Lagynochthoniusdaidaiensis* sp. nov. can be easily distinguished from *L.fengi* Hou, Gao & Zhang, 2022, *L.retrorsus* Hou, Gao & Zhang, 2022, *L.serratus* Hou, Gao & Zhang, 2022, *L.spinulentus* Hou, Gao & Zhang, 2022, *L.xiaolinensis* Hou, Gao & Zhang, 2022 and *L.yaowangguensis* Hou, Gao & Zhang, 2022 by the absence of intercalary teeth on both chelal fingers; from *L.crassus* Hou, Gao & Zhang, 2022 by lacking a pair of anterior eyespots; from *L.magnidentatus* Hou, Gao & Zhang, 2022 and *L.xinjiaoensis* Hou, Gao & Zhang, 2022 by the presence of two carapaceal antero-median setae; from *L.bailongtanensis* Li, Liu & Shi, 2019, *L.minimus* Hou, Gao & Zhang, 2022 and *L.xibaiensis* Hou, Gao & Zhang, 2022 by the number of setae on tergites I–II (2 vs. 3–4) (Li et al. 2019; Hou et al. 2022b).

##### Distribution.

Known only from the type locality.

## Supplementary Material

XML Treatment for Tyrannochthonius

XML Treatment for Tyrannochthonius calvatus

XML Treatment for Tyrannochthonius capito

XML Treatment for Lagynochthonius

XML Treatment for Lagynochthonius daidaiensis
